# Some Observations Relative to Gangrene Resulting from Serious Frost-Bite

**Published:** 1917-11

**Authors:** 


**Some Observations Relative to Gangrene Resulting from Serious Frost-Bite.** Made by M. BERTRAND, aide-major de 1re classe, and reported by Ch. WALTHER. Trans. and abstracted from the *Bulletins et Mémoires de la Société de Chirurgie de Paris*, July 10, 1917.

Of 511 cases of frost-bite gangrene resulted in 60. Almost invariably the causes were cold, dampness, and inactivity. The 60 cases were characterized by massive necrosis complicated with infection. In 40 cases there was an almost complete moist gangrene of the foot. In 35 cases the lesions were bilateral: in one, unilateral. Of 13 cases in which only the front of the foot was affected the lesions were bilateral in 11 and unilateral in 2. In some cases moist and dry gangrene both appeared on different feet or hands of the same person.

At the end of ten days (at the time when most of the cases entered the service) all the special characteristics of freezing had disappeared, giving place to septic moist gangrene.

Points worthy of note:

1. Slight local pain compared with the usual suffering from freezing.

2. The persistence in cases of total gangrene of a V-shaped flap of healthy skin extending on the inside of the foot, to the level of the plantar arch — due probably to the greater vascularity of this region.

In most cases evolution was regular under proper treatment. In a few days high temperature was perceptibly lowered. Urine, slight at first, became abundant, albuminuria decreased, and the general condition improved. In some cases after about 8 days of apyrexia, a rise in temperature and the return of edema indicated an infection of the articulations of the foot.

The most dreaded complication was tetanus which appeared in

5 cases that were least serious in appearance. In spite of preventive injections of serum, it set in about twelve days after the freezing and developed in an acute form causing early death — in one case, within 18 hours.

In 2 cases a gaseous secondary gangrene appeared and extended within a few hours to above the knee making a thigh amputation necessary. In one case death resulted after a slight improvement. In the other, recovery followed the amputation of both feet.

The serum secreted from the infected tissues of these two patients when injected into guinea pigs resulted in the development of typical gas gangrene.

In 18 cases of moist gangrene gas developed, due not to true gaseous gangrene, however, but to putrefaction and decomposition. One case succumbed to septicemia.

Dissection of the amputated members showed a putrid deliquescence at the bottom of the foot and an unrecognizable condition of the tissues. No conclusion could be drawn as to the cause of the unfavorable developments.

Disinfection of the septic gangrenous regions was invariably attempted in deep incisions by means of the thermo-cautery and the use of such chemicals as permanganate, ether, iodoform, and formol. Gomenol oil bandages (lanoline, 500; oil, 500; gomenol, 50) gave good results. Disinfection had to be continued until the complete limitation of necrosed parts permitted amputation, except when conditions did not permit of the delay. 18 amputations (8 bilateral) were made.

The amputations were generally either of a low circular variety at the line of demarcation or amputations of Guyon. In the latter the interior preserved flap and sometimes a posterior flap were utilized. In many precarious cases spinal anesthesia was employed. Shock was comparatively slight, and improvement followed within two days. The authors advise a delay before operating to permit spontaneous limitation in order to preserve a maximum of tissue.

They conclude that in the 60 gangrenous cases the seriousness was due less to the element of freezing than to that of infection. They therefore recommend the most energetic disinfection from the start and every possible precaution against sepsis.

